# Untargeted Metabolomics Identifies Faecal Filtrate-Derived Metabolites That Disrupt *Clostridioides difficile* Metabolism and Confer Gut Barrier Cytoprotection

**DOI:** 10.3390/ijms262211221

**Published:** 2025-11-20

**Authors:** Fatimah I. Qassadi, Charlotte Johnson, Karen Robinson, Ruth Griffin, Christos Polytarchou, Dina Kao, Dong-Hyun Kim, Rian L. Griffiths, Zheying Zhu, Tanya M. Monaghan

**Affiliations:** 1 School of Pharmacy, Prince Sattam bin Abdulaziz University, Al-Kharj 11942, Saudi Arabia; 2 Nottingham Digestive Diseases Centre, Translational Medical Sciences Unit, School of Medicine, University of Nottingham, Nottingham NG7 2UH, UK; 3 Biodiscovery Institute, School of Medicine, University of Nottingham, Nottingham NG7 2RD, UK; 4 NIHR Nottingham Biomedical Research Centre, University of Nottingham, Nottingham NG7 2UH, UK; 5 Vaccines and Therapeutics Group, Biodiscovery Institute, School of Life Sciences, University of Nottingham, Nottingham NG7 2RD, UK; 6 Department of Biosciences, Centre for Systems Health and Integrated Metabolic Research (SHiMR), Nottingham Trent University, Nottingham NG11 8NS, UK; 7 John van Geest Cancer Research Centre, School of Science & Technology, Nottingham Trent University, Nottingham NG11 8NS, UK; 8 Division of Gastroenterology, Department of Medicine, Faculty of Medicine and Dentistry, University of Alberta, Edmonton, AB T6G 2R3, Canada; 9 School of Pharmacy, University of Nottingham, Nottingham NG7 2RD, UK

**Keywords:** bacterial infection, enteric pathogens, faecal microbiota transplantation, faecal filtrate, antimicrobial resistance, *Clostridioides difficile*

## Abstract

Recurrent *Clostridioides difficile* infection (rCDI) remains a major therapeutic challenge. Although faecal microbiota transplantation (FMT) is highly effective and thought to restore microbial composition and metabolic function, the mechanisms underlying its success are not fully understood. In particular, the contribution of non-bacterial components such as soluble metabolites remains unclear. Therefore, further investigation is needed to identify the mechanistic drivers of FMT efficacy and clarify how non-bacterial factors contribute to therapeutic outcomes. Here, we applied untargeted three-dimensional Orbitrap secondary ion mass spectrometry (3D OrbiSIMS) to profile faecal metabolic reprogramming in rCDI patients pre- and post-FMT, alongside *C. difficile* cultures exposed to sterile faecal filtrates. FMT induced extensive metabolic shifts, restoring glyoxylate/dicarboxylate and glycerophosphoinositol pathways and normalising disrupted bile acid and amino acid profiles. Faecal filtrate exposure caused strain-specific metabolic disruption in *C. difficile*, depleting proline, fumarate and succinate while enriching tryptophan. While multiple metabolite classes were profiled, the most significant functional changes were observed in lipids. Lipidomics identified >3.8-fold enrichment of phosphatidylinositol (PI) species, which localised to bacterial membranes and conferred cytoprotection against *C. difficile* toxins and other epithelial insults. Spatial metabolomics imaging revealed, for the first time, metabolite compartmentalisation within *C. difficile*, with proline and succinate broadly distributed across the cell surface and fumarate confined to distinct microdomains, highlighting functional heterogeneity in pathogen metabolism. Collectively, these findings demonstrate that soluble metabolites within faecal filtrates mediate pathogen suppression and epithelial barrier protection, establishing metabolite-driven mechanisms underlying FMT efficacy and identifying PI lipids as candidate post-biotic therapeutics for rCDI.

## 1. Introduction

Recurrent *Clostridioides difficile* infection (rCDI) remains a major clinical challenge, driven by antibiotic-associated disruption of the gut microbiota [1]. Faecal microbiota transplantation (FMT) is highly effective in resolving rCDI by restoring microbial diversity, yet its full mechanisms of action remain poorly understood [2,3]. Beyond bacterial engraftment, non-bacterial components such as bacteriophages and metabolites appear to contribute to therapeutic benefit [4]. Notably, a small, open-label trial using sterile faecal filtrates devoid of live bacteria successfully prevented *C. difficile* infection recurrence in five patients, suggesting that soluble bioactive compounds may support recovery [5]. More recently, a multicentre, double-blinded trial comparing lyophilised donor stool (LFMT) with lyophilised sterile faecal filtrate (LSFF) found that LSFF was less effective (65% vs. 88% recurrence-free at 8 weeks) and did not fully restore key commensal taxa, confirming the importance of viable microbes to optimal efficacy [6]. Nonetheless, the partial efficacy in preventing rCDI observed with LSFF suggests a complementary role for non-bacterial factors, including metabolites, in disease resolution. Alongside pathogen suppression, restoration of gut barrier integrity is critical in rCDI, as toxin-mediated epithelial damage drives inflammation and relapse [7]. Identifying metabolites with cytoprotective activity therefore has significant translational potential.

Metabolomics provides a powerful lens to capture the functional outputs of host–microbe interactions [8,9]. While traditional platforms like Liquid Chromatography-Mass Spectrometry (LC-MS) and Nuclear Magnetic Resonance (NMR) offer valuable insights, they lack the spatial information, sensitivity, or metabolite breadth required to capture the fuller picture of treatment-associated changes in complex samples such as stool [10,11]. Mass spectrometry imaging, however, offers the opportunity to spatially map ions of interest in an untargeted and label-free manner. Previously, bacterial metabolites, lipids and proteins have been studied, and surface-sampling and MS imaging approaches have been summarised [12]. Among the available modalities, three-dimensional Orbitrap secondary ion mass spectrometry (3D OrbiSIMS) is an emerging technology that enables spatially resolved metabolic profiling with minimal sample preparation and broad metabolite coverage [9,13].

Here, we applied 3D OrbiSIMS to characterise faecal metabolic signatures in patients with rCDI treated with FMT (delivered via colonoscopy or oral capsules) [14]. By analysing pre-treatment, post-treatment, and donor-derived samples themselves, alongside *C. difficile* cultures co-incubated with faecal filtrates from FMT donors, we aimed to (i) define metabolic signatures associated with clinical resolution of rCDI, (ii) elucidate mechanisms by which faecal filtrates modulate *C. difficile* energy and lipid metabolism, and (iii) functionally test candidate metabolites for cytoprotective effects on the gut epithelial barrier [5,15]. Our findings reveal distinct shifts in amino acid, bile acid, and lipid pathways, as well as faecal filtrate-derived lipids with cytoprotective activity that preserve gut epithelial barrier integrity. Together, these results provide mechanistic insights into how soluble metabolites contribute to the therapeutic success of FMT.

## 2. Results

### 2.1. Distinct Metabolomic Profiles in Pre-FMT, Post-FMT, and Donor Samples by OrbiSIMS

To ensure clarity in sample grouping and analysis, the dataset consisted of faecal samples collected from 12 rCDI patients at two timepoints (Pre-FMT and Post-FMT; 24 biological samples total) and from 4 healthy donors (4 biological samples), resulting in 28 biological samples. Each biological sample was analysed in triplicate using OrbiSIMS to account for technical variability, generating 36 processed spectra in total after quality filtering. For multivariate analyses, technical replicates were averaged to prevent over-representation of individual samples. Thus, the PCA was constructed using 18 datapoints, representing one averaged spectrum per biological sample (12 patient samples and 4 donor samples across the two timepoints). This approach preserves biological variation while controlling for technical noise.

In total, 36 faecal filtrate samples (pre-FMT, post-FMT, and donor, each in triplicate) were analysed. OrbiSIMS ion spectra revealed 2532 peaks in negative polarity and 4847 in positive polarity, representing a broad spectrum of metabolites and organic compounds. Using SIMS-MFP (v1.1, MATLAB R2023a), 1571 metabolites were confidently identified across all samples.

**Figure 1:**
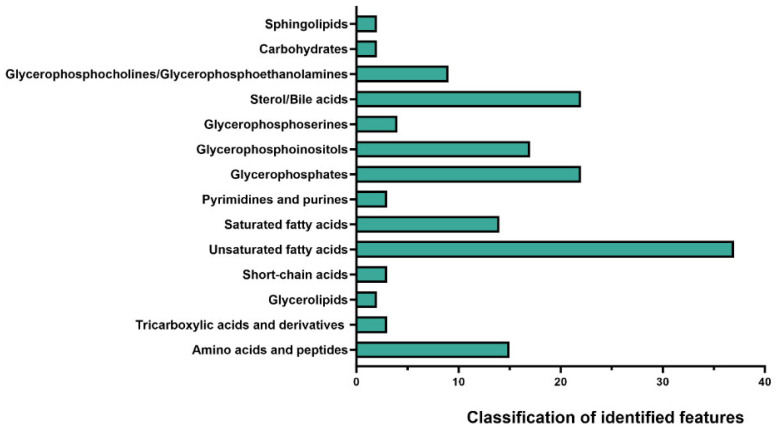
Classification of metabolic features in faecal filtrates. 3D OrbiSIMS analysis of faecal samples from pre-FMT, post-FMT, and donor groups revealed broad coverage across metabolites. The results represent the aggregate of all stages, with unsaturated fatty acids accounting for the largest group of identified features, followed by glycerophosphocholines/glycerophosphoethanolamines and sterol/bile acids.

Figure 1 presents the overall classification of metabolic features across all sample groups (pre-FMT, post-FMT, and donors), summarising the aggregate metabolic diversity detected by 3D OrbiSIMS. A broad range of metabolites was identified in a single acquisition, including amino acids, bile acids, short-chain fatty acids, nucleobases, glycerophospholipids, and sugars, illustrating the extensive metabolic diversity present in FMT samples.

**Figure 2:**
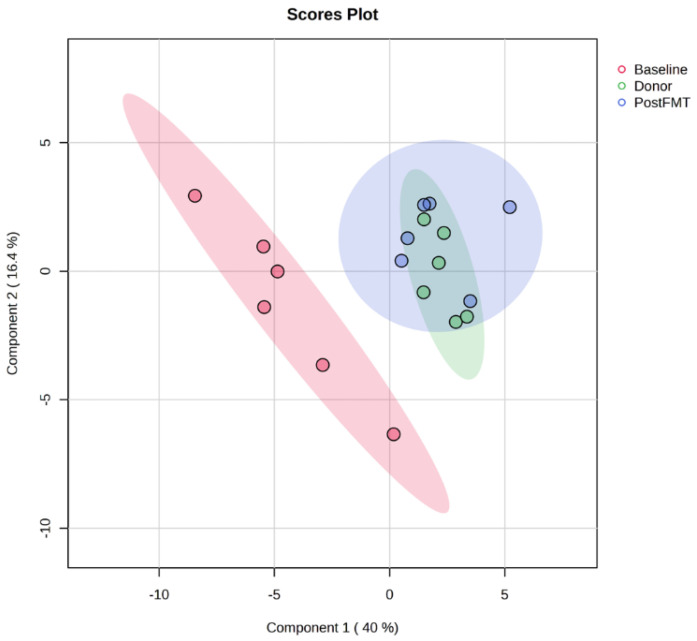
Principal Component Analysis (PCA) of faecal metabolomes pre- and post-FMT. PCA scores plot of OrbiSIMS spectra from donor and recipient stool samples at baseline (pre-FMT, red) and 12 weeks post-FMT (blue), and donors (green). Pre-FMT samples formed a distinct cluster, while post-FMT samples overlapped substantially with donors, indicating that FMT restored the disrupted gut metabolome in rCDI patients. FMT denotes faecal microbiota transplantation, rCDI recurrent *Clostridioides difficile* infection.

**Figure 3:**
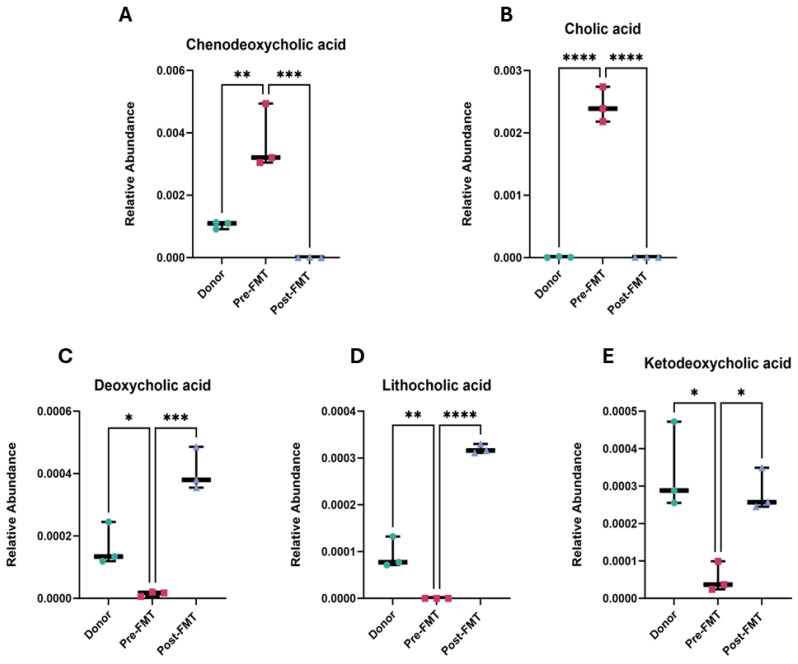
Altered bile acid profiles in rCDI patients pre- and post-FMT. Relative abundances of major primary and secondary bile acids were quantified in donor stool, patient samples prior to FMT (Pre-FMT), and patient samples following FMT (Post-FMT). (**A**) Chenodeoxycholic acid, (**B**) cholic acid, (**C**) deoxycholic acid, (**D**) lithocholic acid, and (**E**) ketodeoxycholic acid. Each point represents an individual sample, and bars indicate mean ± SEM. Each point represents one biological sample, where technical OrbiSIMS replicates were averaged prior to analysis. Statistical significance was determined using a one-way ANOVA with Tukey’s multiple comparison post hoc test. Significance is indicated as follows: * *p* < 0.05, ** *p* < 0.01, *** *p* < 0.001, **** *p* < 0.0001. Analyses were performed in GraphPad Prism v10. FMT denotes faecal microbiota transplantation, rCDI recurrent *Clostridioides difficile* infection.

Post-FMT samples displayed a clear shift in metabolic composition toward that of donor stool. Violin plots (Supplementary Figure S1) demonstrated a general trend toward normalisation of metabolite abundance following treatment. Principal Component Analysis (PCA) further confirmed this effect (Figure 2): pre-FMT samples clustered distinctly apart from post-FMT and donor samples, whereas post-FMT samples overlapped substantially with donors. This convergence indicates that FMT clusters restore the disrupted gut metabolome. The marked shifts in metabolic profiles across treatment stages are presented in Figure 3.

### 2.2. Elevated Primary Bile Acids and Amino Acids in Pre-FMT Stool Samples

Comparison of pre- and post-FMT metabolomes identified 316 metabolites with significant changes in abundance (FDR-adjusted *p* < 0.05, FC > 2, VIP > 1). Among these, bile acid metabolism showed the most striking observations (Figure 3). Pre-FMT samples exhibited elevated levels of primary bile acids, including chenodeoxycholic acid (*p* < 0.001) and cholic acid (*p* < 0.0001). In contrast, secondary bile acids, deoxycholic acid, lithocholic acid, and ketodeoxycholic acid were significantly depleted (*p* < 0.001, *p* < 0.0001, and *p* < 0.05, respectively).

### 2.3. Enrichment of Amino Acids in Pre-FMT Samples

Pre-FMT stool samples showed significantly elevated levels of amino acids including proline, tryptophan, lysine, and phenylalanine (*p* < 0.001) compared to post-FMT samples (Table 1). This enrichment is consistent with previous reports that amino acid biosynthesis is upregulated in CDI, supporting *C. difficile* proliferation. Proline was particularly elevated, showing a 49.8-fold increase in pre-FMT compared with post-FMT samples.

### 2.4. Pathway Analysis of FMT-Induced Metabolic Reprogramming

Pathway enrichment analysis revealed significant alterations in several key metabolic pathways following FMT, particularly those involving small molecules and lipids. The most notable shifts were observed in glyoxylate and dicarboxylate metabolism alsoglycerophosphoinositol (PI) metabolism (Table 2). Additional changes were detected in amino acid biosynthesis pathways, including branched-chain and aromatic amino acid metabolism. These findings underline the potential of FMT to restore metabolic homeostasis by re-balancing fundamental energy and lipid pathways. The two most strongly affected pathways, glyoxylate/dicarboxylate and glycerophosphoinositol metabolism, are explored in detail in the following sections.

### 2.5. FMT Alters Glyoxylate and Dicarboxylate Metabolism in rCDI Patients

**Figure 4:**
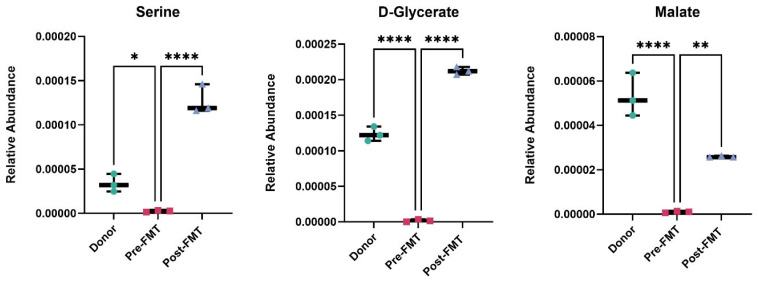
Restoration of glyoxylate and dicarboxylate metabolism following FMT. Relative abundance of serine, D-glycerate, and malate in stool samples from rCDI patients pre-FMT, post-FMT, and healthy donors, measured by OrbiSIMS. FMT restored metabolite levels toward those of donors, indicating recovery of glyoxylate and dicarboxylate metabolism. Data are shown as mean ± SD. One-way ANOVA with Tukey’s post hoc test was used for group comparisons. Each point represents one biological sample, where technical OrbiSIMS replicates were averaged prior to analysis. Statistical significance is represented as follows: * *p* < 0.05, ** *p* < 0.01, **** *p* < 0.0001. Analyses were performed in GraphPad Prism v10. FMT denotes faecal microbiota transplantation.

Metabolomic profiling revealed significant modulation of glyoxylate and dicarboxylate metabolism after FMT. Post-FMT stool samples showed a 26.7-fold increase in malate, a 1.9-fold increase in the amino acid serine (a microbial metabolite produced by *Lactobacillus* spp. and *Bacteroides* spp.), and a 1.6-fold increase in D-glycerate (Figure 4).

### 2.6. Restoration of Phosphatidylinositol Lipid Metabolism After FMT

**Figure 5:**
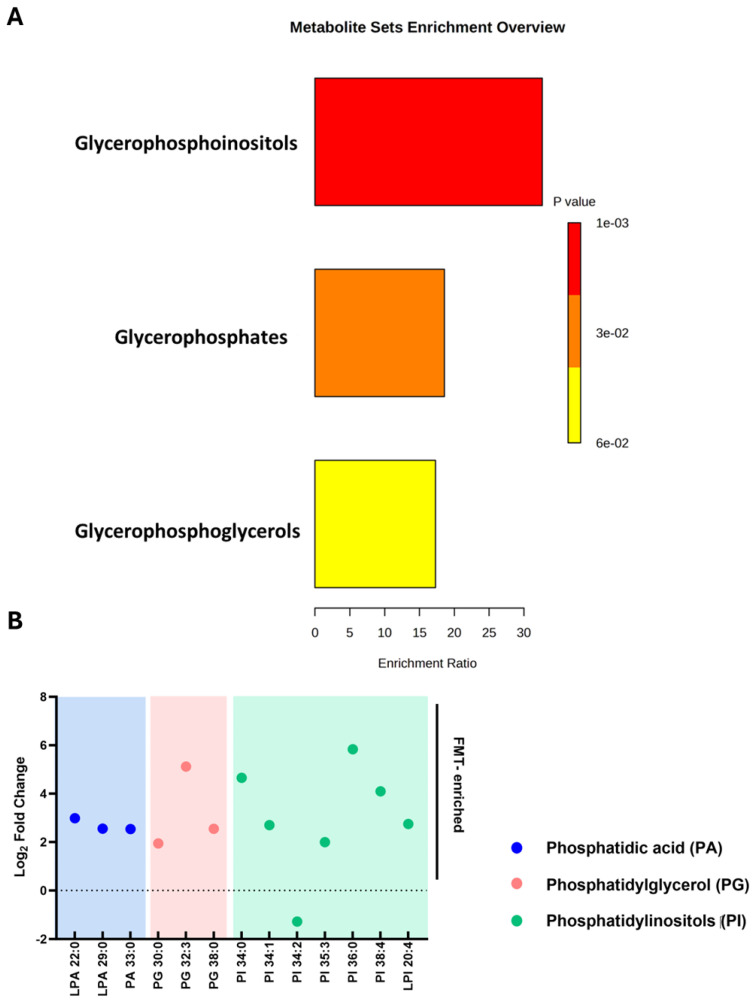
Enrichment and fold-change analysis of lipid species following FMT in patients with rCDI. (**A**) Metabolite set enrichment analysis of phosphatidylinositols (PI), phosphatidic acid (PA), and phosphatidylglycerol (PG) post-FMT. Bar length represents enrichment ratio, while colour denotes significance (*p*-value), with red representing higher significance. (**B**) Log fold changes in lipid species, highlighting increased abundance in PA (blue), PG (pink), and PI (green) lipids. Only lipid species that were significantly altered following FMT (adjusted *p* < 0.05; fold-change > 2) are displayed. The dashed line at 0 indicates no change, values above the line indicate an increase, and values below indicate a decrease. Lipid species are grouped and colour-coded by class. Analyses were performed using GraphPad Prism v10. FMT denotes faecal microbiota transplantation, rCDI recurrent *Clostridioides difficile* infection.

FMT induced significant changes in glycerophospholipid metabolism, particularly within the phosphatidylinositol (PI) subclass. Post-FMT samples exhibited increased abundance of PI, phosphatidic acid (PA), and phosphatidylglycerol (PG) lipid species, indicating targeted modulation of lipid metabolic pathways (Figure 5A,B). Several PI lipid species showed marked log-fold increases (FC > 2, *p* < 0.001), indicating enhanced lipid metabolic activity and suggesting a role of PI lipids in the therapeutic reprogramming of the rCDI metabolome.

### 2.7. Faecal Filtrate Treatment Induces Strain-Specific Metabolic Reprogramming in C. difficile

**Figure 6:**
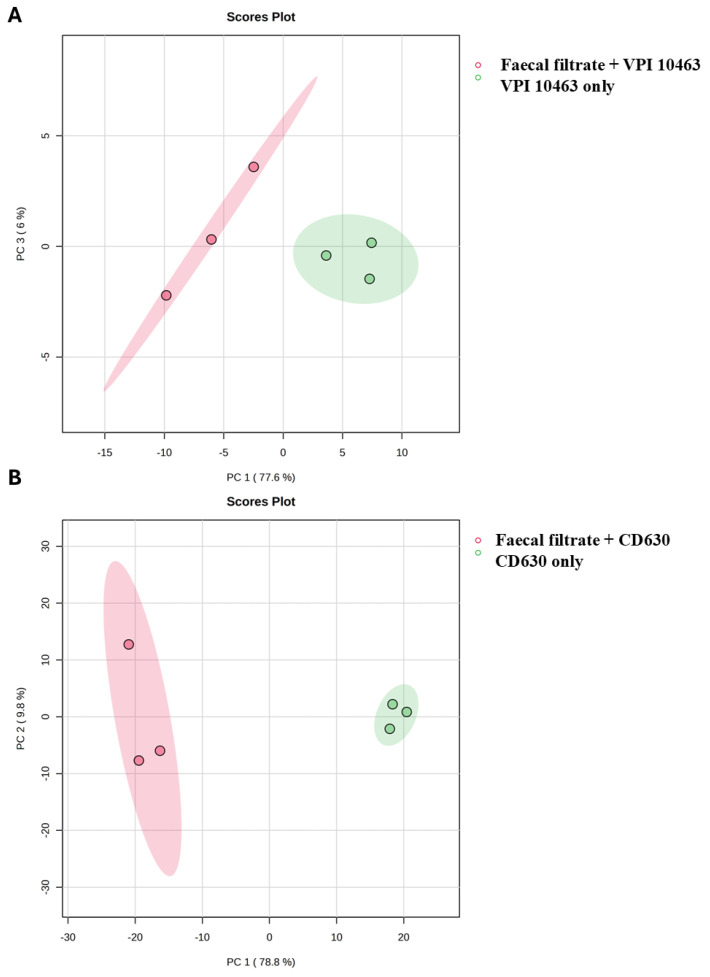
PCA of *C. difficile* metabolomes following faecal filtrate treatment. Principal Component Analysis (PCA) score plots illustrating metabolic profiles of untreated and faecal-filtrate-treated *C. difficile* strains (**A**) VPI 10463 and (**B**) CD630. Clear separation between the two groups suggests distinct, strain-specific metabolic reprogramming induced by faecal filtrates.

While bile acids and short-chain fatty acids (SCFAs) are known mediators of FMT’s efficacy, the broader metabolomic impact of faecal filtrates on *C. difficile* metabolism has remained unclear. To address this, untargeted 3D OrbiSIMS metabolomic profiling was conducted on two toxigenic *C. difficile* strains, VPI 10463 and CD630, comparing untreated controls with samples treated using sterile faecal filtrates derived from healthy FMT donors. Across positive and negative ion modes, 3516 chemical features were detected. Principal Component Analysis (PCA) revealed clear separation between untreated and filtrate-treated samples for both strains (Figure 6A,B), confirming that faecal filtrate exposure drives substantial and strain-specific metabolic shifts. These findings suggest that soluble metabolites present in faecal filtrates directly modulate *C. difficile* metabolic activity, potentially contributing to the clinical efficacy of FMT in rCDI.

### 2.8. Strain-Specific Metabolic Responses in VPI 10463 and CD630

**Figure 7:**
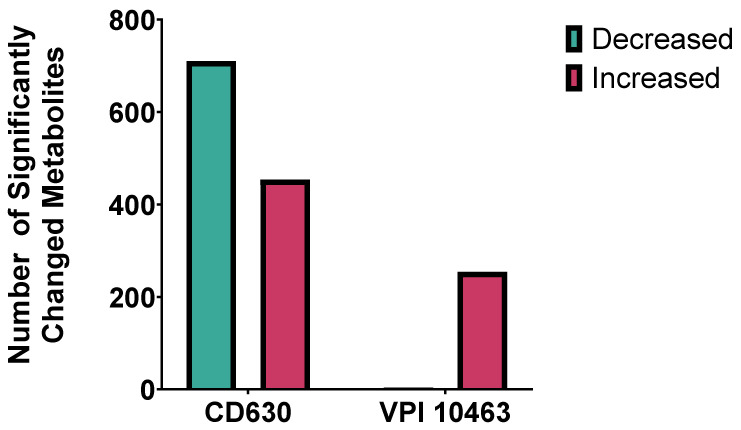
Strain-specific metabolic responses of *C. difficile* to faecal filtrate. Comparison of significantly altered metabolites in *C. difficile* strains CD630 and VPI 10463 co-cultured with faecal filtrates from donor stool. CD630 displayed a broader response, with ~700 metabolites decreased and ~300 increased, whereas VPI 10463 primarily showed ~200 increased. Significance was defined as *p* < 0.05.

Co-culture of *C. difficile* with faecal filtrates demonstrated marked strain-dependent differences in metabolic response. The highly toxigenic VPI 10463 strain showed relatively few metabolomic changes, with 254 metabolites significantly increased and only 4 decreased (*p* < 0.05, FC > 2, VIP > 1). By contrast, CD630 exhibited a much broader response, with 454 metabolites increased and 710 decreased under identical conditions (Figure 7). These findings highlight distinct metabolic susceptibilities between strains, suggesting that filtrate-derived metabolites exert variable effects depending on *C. difficile* genotype.

### 2.9. Faecal Filtrate Co-Culture Alters Lipid Metabolism in C. difficile

**Figure 8:**
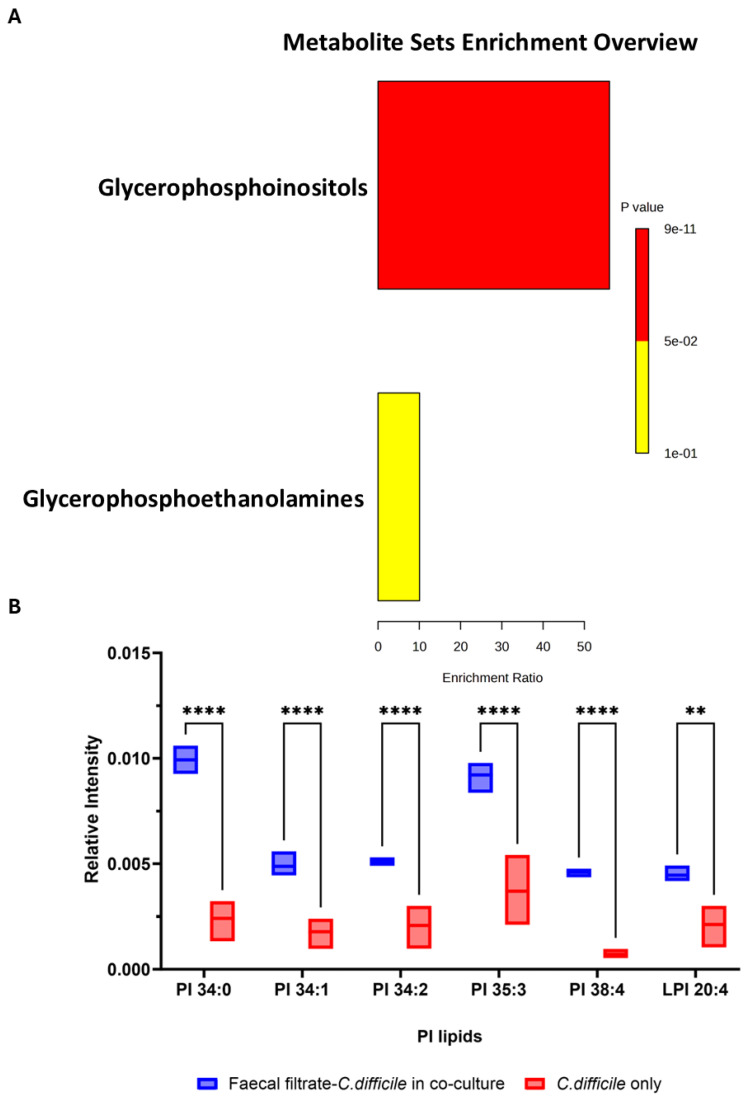
Enrichment analysis and relative intensities of key lipid metabolite changes following faecal filtrate exposure. (**A**). Pathway enrichment analysis of significantly altered metabolites in VPI 10463 and CD630 revealed the highest enrichment for glycophosphoinositols (*p* < 0.001). (**B**) Relative intensity of specific putative PI lipid species identified in VPI 10463 strain, including PI 34:0 (*m*/*z* = 838.5571, C_43_H_83_O_13_P^−^), PI 34:1 (*m*/*z* = 835.5353, C_43_H_80_O_13_P^−^), PI 34:2 (*m*/*z* = 833.5185, C_43_H_78_O_13_P^−^), PI 35:3 (*m*/*z* = 845.5185, C_44_H_78_O_13_P^−^), PI 38:4 (*m*/*z* = 885.5498, C_47_H_82_O_13_P^−^), and LPI 20:4 (*m*/*z* = 619.2889, C_29_H_84_O_12_P^−^) post-faecal filtrate treatment, with statistical significance (** *p* < 0.01, **** *p* < 0.0001).

Metabolomic profiling of *C. difficile* strains VPI 10463 and CD630 co-cultured with faecal filtrates revealed significant changes in lipid metabolites. Pathway enrichment analysis identified a marked enrichment in glycerophospholipid metabolism, particularly within the glycophosphoinositol subclass (*p* < 0.001) (Figure 8A).

Among the most altered lipid groups, phosphatidylinositol (PI) lipids exhibited marked increases in abundance in VPI 10463 following faecal filtrate treatment. Specific PI lipid species, including PI 34:0, PI 34:1, PI 34:2, PI 35:3, PI 38:4, and LPI 20:4, showed >3.8-fold increases, with high statistical significance (** *p* < 0.01, **** *p* < 0.0001) (Figure 8B). These results suggest that metabolites from faecal filtrate modulate lipid pathways directly linked to bacterial physiology and virulence.

### 2.10. Faecal Filtrate Modulates Virulence-Associated Metabolites in C. difficile

**Figure 9:**
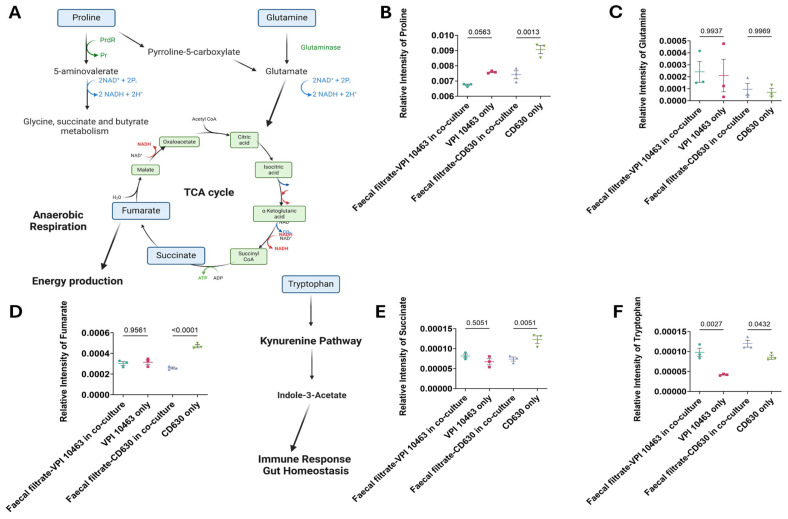
Pathway mapping of metabolites altered in faecal filtrate-treated *C. difficile.* (**A**) Schematic illustration of metabolic pathways supporting *C. difficile* survival and virulence. Proline is converted into pyrroline-5-carboxylate, fuelling the TCA cycle via fumarate under anaerobic respiration. Glutamine is metabolised to glutamate, which also contributes to TCA cycle energy metabolism. Tryptophan enters the kynurenine pathway, inducing indole-3-acetate, a metabolite implicated in immune regulation and gut homeostasis. (**B**–**F**) Relative abundances of proline, glutamine, fumarate, succinate, and tryptophan in *C. difficile* cultures with and without faecal filtrate treatment. Data are presented as mean  ±  SD with individual data points shown. One-way ANOVA followed by Tukey’s multiple comparison test was used for group comparisons (*p* < 0.05 considered significant). PrdR = proline-dependent regulatory protein, Pr = proline reductase. Spatial Metabolic Features Identified by 3D OrbiSIMS Imaging.

To evaluate how faecal filtrate influences metabolites associated with *C. difficile* virulence, key metabolic compounds were quantified in treated and untreated cultures of toxigenic strains VPI 10463 and CD630. Co-culture with faecal filtrates resulted in reduced proline levels in both strains, with a statistically significant decrease in CD630 (*p* = 0.0013) (Figure 9B). Proline remained elevated in untreated cultures, consistent with its role as an energy source in Stickland fermentation. Glutamate levels were unchanged across all conditions (*p* = 0.99) (Figure 9C). In contrast, fumarate and succinate, two intermediates of the TCA cycle, were significantly reduced in faecal filtrate-treated samples compared to untreated controls. Fumarate levels showed a highly significant decrease (*p* < 0.0001), while succinate levels were also significantly lower (*p* = 0.005) in both strains following treatment (Figure 9D). In addition, tryptophan levels were significantly elevated in co-cultured samples compared to untreated controls. This increase was observed in both strains, with *p* = 0.0027 for VPI 10463 and *p* = 0.0432 for CD630 (Figure 9E), consistent with activation of the immunoregulatory pathways.

**Figure 10:**
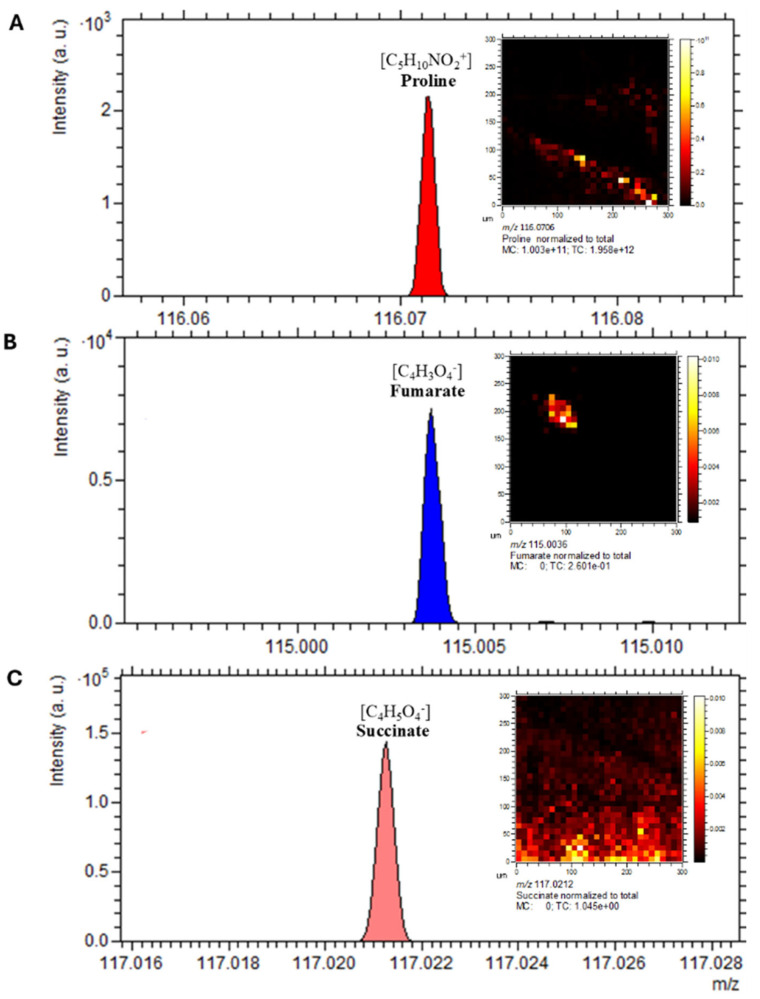
Spatial distribution of virulence-associated metabolites in *C. difficile*. The spectrum and distribution of annotated discriminative ions from the 3D OrbiSIMS profiling analysis visualised using Orbitrap depth profiling and imaging of a *C. difficile* sample. (**A**) proline at *m*/*z* 116.0706, (**B**) fumarate at *m*/*z* 115.0036, (**C**) succinate at *m*/*z* 117.0212. Scale bar is 20 µm.

Gas cluster ion beam (GCIB) 3D OrbiSIMS imaging revealed spatial localisation of key metabolites within *C. difficile* cells, including fumarate, succinate, and proline, all of which have established roles in *C. difficile* metabolism and virulence (Figure 9). Proline and succinate exhibited broad distribution patterns across the analysed cell surface, with localised regions of high intensity, whereas fumarate exhibited a more concentrated and restricted spatial pattern (Figure 10A–C). These findings highlight heterogeneity in metabolite distribution, suggesting compartmentalised metabolic activity within *C. difficile.*

### 2.11. Spatial Localisation of Lipid Species in C. difficile

**Figure 11:**
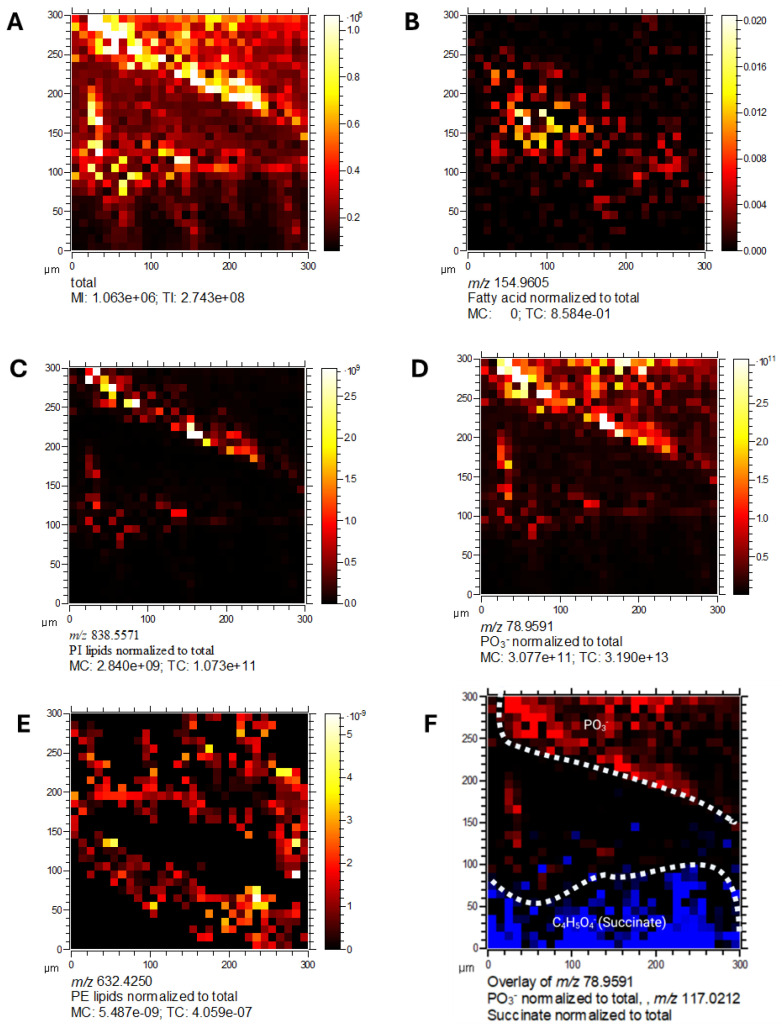
Spatial localisation of lipid species in *C. difficile* by 3D OrbiSIMS imaging. Mass spectrometry imaging of *C. difficile* cells showing. (**A**) Total ion counts, (**B**) fatty acids (*m*/*z* 154.9005), (**C**) PI lipids (*m*/*z* 838.5571), (**D**) PO_3_^−^ (*m*/*z* 78.9591), (**E**) PE lipids (*m*/*z* 632.4250), (**F**) overlay of PO_3_^−^ (*m*/*z* 78.9591) and succinate (*m*/*z* 117.0212). Lipid classes were dispersed throughout the bacterial sample, consistent with their role in membrane structure.

Mass spectrometry imaging of lipid species revealed clear spatial localisation of bacterial membrane-associated phospholipids. Detected species included fatty acids (*m*/*z* 154.9005), PI lipids (*m*/*z* 838.5571), PO_3_^−^ (*m*/*z* 78.9591), and PE lipids (*m*/*z* 632.4250) (Figure 11A–E). These lipid classes were dispersed throughout the bacterial sample, consistent with their roles as key structural and functional components of cell membranes.

### 2.12. Metabolic Compartmentalisation Revealed by Spatial Imaging

Overlay analysis of PO_3_^−^ and succinate (Figure 11F) showed distinct and non-overlapping spatial distributions, suggesting these metabolites occupy separate cellular niches. This spatial segregation indicates metabolic compartmentalisation within *C. difficile*, consistent with specialised functional roles. Collectively, these imaging results highlight the power of 3D OrbiSIMS for high-resolution spatial mapping of bacterial metabolomes, revealing heterogeneity in the localisation of lipids, intermediates, and virulence-associated metabolites with *C. difficile* cells.

### 2.13. Metabolite-Specific Cytoprotective Effects of Phosphatidylinositols Against Toxin-Induced Epithelial Barrier Damage

**Figure 12:**
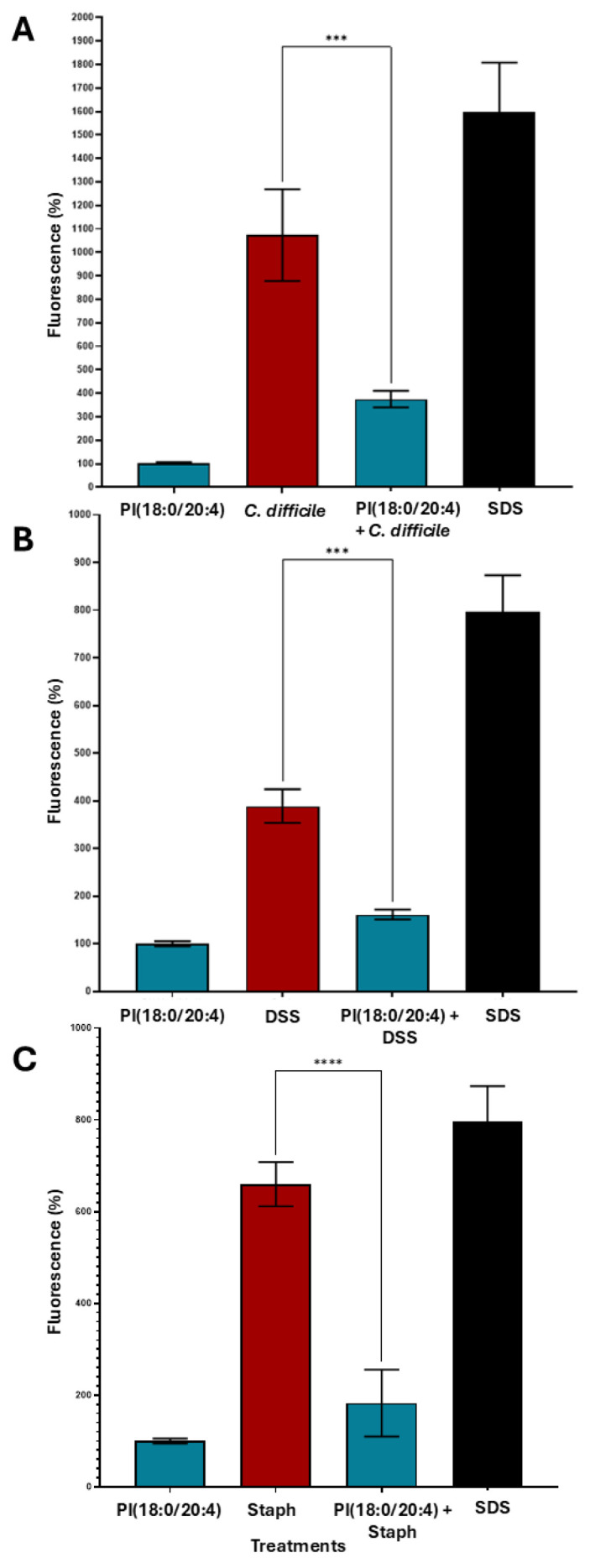
Cytoprotective effects of PI (18:0/20:4) against various epithelial inflammatory insults. Caco-2 cells (~5000 cells/well) were seeded in biological triplicates (n = 3) into Transwells. After 2 weeks of differentiation, epithelial barrier integrity was confirmed by FITC-dextran measurements. Cells were treated with PI (18:0/20:4). After 15 min, cells were exposed to the respective toxigenic epithelial insults. Fluorescence was measured after 48 h or 96 h to assess membrane damage. (**A**) PI (18:0/20:4) mitigated epithelial damage induced by *C. difficile* toxins TcdA and TcdB at 96 h. (**B**) PI (18:0/20:4) conferred protection against DSS-induced barrier damage at 48 h. (**C**) PI (18:0/20:4) significantly reduced epithelial injury caused by *Staphylococcus aureus* enterotoxin B at 96 h. Data are presented as mean ± SEM. Asterisks denote statistical significance: *** *p* < 0.001, **** *p* < 0.0001.

The observed metabolomic alterations prompted further investigation into the functional relevance of specific lipids enriched during faecal filtrate co-culture. Among the significantly upregulated lipid species, several glycerophospholipids, including PIs, were consistently elevated. To determine whether these lipids conferred any biological activity on host cells, selected metabolites were assessed for their cytoprotective potential in human intestinal epithelial barrier models subjected to bacterial toxin insults (Figure 12). Among the tested lipid metabolites, PI (18:0/20:4) demonstrated notable cytoprotective effects under specific toxin-induced insults. In Caco-2 Transwell models, treatment with PI (18:0/20:4) significantly preserved intestinal epithelial barrier integrity at 96 h post-exposure to *C. difficile* toxins A (TcdA) and B (TcdB) (*p* < 0.0001), and at 48 h in response to dextran sulfate sodium (DSS) insult (*p* < 0.001) (Figure 12A,B). Additionally, protective effects were observed at 96 h following *Staphylococcus aureus* enterotoxin B challenge (Figure 12C). In contrast, neither PI (16:0/18:1(9Z) nor LPI (20:4/0:0) conferred gut barrier protection under any tested condition, including *C. difficile* toxins A and B, DSS and *Pseudomonas aeruginosa* challenges (Supplementary Figure S2). These findings suggest a metabolite-specific and time-dependent cytoprotective effect.

## 3. Discussion

Using 3D OrbiSIMS metabolomics, applied here for the first time in this context, we identified distinct metabolic reprogramming following rCDI resolution, including enrichment of phosphatidylinositol (PI) lipids, providing new insight into the molecular mechanisms by which faecal filtrates restore the gut microbial ecosystem. Multiple in vitro, ex vivo, animal model and human studies have demonstrated an alteration in bile acid milieu in CDI; specifically, an enrichment of primary conjugated bile acids (including taurocholic acid, which promotes germination of *C. difficile* spores) and loss of secondary bile acids (which inhibit the growth of *C. difficile* and may bind to and limit activity of *C. difficile* toxins) [16,17]. The multiple rounds of broad-spectrum antibiotics received prior to FMT likely disrupted commensal bacteria responsible for converting primary to secondary bile acids, resulting in elevated primary bile acid levels that promote *C. difficile* germination and recurrence [18]. Our findings further support the role of lipids as central mediators in CDI pathogenesis and treatment responses. Whereas earlier studies observed global lipid depletion, particularly of cholesteryl esters and linoleic acid, with partial recovery following antibiotics and greater restoration after FMT [19], our data reveal a complementary and distinct pattern. Specifically, PI lipid enrichment not only reflects metabolic reprogramming but also contributes directly to gut epithelial barrier protection.

Interestingly, some metabolite levels post-FMT were observed to exceed those measured in donor samples. This likely reflects differences in the baseline metabolic states of rCDI patients and healthy donors, as well as individual-specific factors including residual microbiota composition, diet, and host metabolic responses [20,21]. Such variations are expected given the dynamic recovery of the gut ecosystem following FMT and highlight that post-treatment metabolite levels may not always mirror donor profiles exactly, but still contribute to restoring overall gut homeostasis.

Restoration of secondary bile acids through FMT primarily creates a metabolically unfavourable environment for *C. difficile*, limiting pathogen fitness. These observations are supported by studies from Mullish and colleagues, who demonstrated that restoration of secondary bile acids and bile salt hydrolase activity via FMT is critical for inhibiting *C. difficile* growth and reducing recurrence [17,22]. Importantly, PI lipids provide host-directed benefits by stabilising the epithelial barrier and modulating inflammatory responses. This dual mechanism reinforces the concept that microbiome-based therapies act through shared microbial and host metabolic networks, rather than targeting a single pathway, highlighting lipid metabolic remodelling as a central feature of CDI, with different lipid classes contributing to distinct aspects of host-microbiome interaction [23,24].

The human gut bacterium *Bacteroides thetaiotaomicron* has been associated with PI lipid production [25], and other commensals, including *Bacteroides fragilis* and *Clostridium* spp., also contribute to glycerophospholipid metabolism [26]. This suggests that FMT restores a bacterial consortium responsible for key metabolic functions in the gut ecosystem. PI lipids are critical mediators of cellular signalling that regulate growth, survival, and inflammation [27]. In murine models of endotoxemia, PI treatment reduced TNF-α synthesis, supporting its anti-inflammatory potential [28], consistent with human data showing that FMT reduces inflammatory markers [29,30]. Together, these findings highlight the therapeutic implications of FMT-induced glycerophospholipid remodelling for host inflammatory responses.

Our data also support the concept that faecal filtrates recapitulate aspects of full FMT by delivering soluble, microbe-derived metabolites. PI lipids showed >3.8-fold enrichment in the toxigenic VPI 10463 strain, paralleling increases observed in post-FMT patient samples. These molecules influence membrane signalling, immune modulation, and epithelial protection, potentially reducing *C. difficile* germination, growth, and toxin production [29,30]. By contrast, acylcarnitines, previously linked to inflammation and dysbiosis [31], were not enriched, suggesting that PI lipids may represent positive biomarkers of FMT efficacy.

In addition to lipid metabolism, faecal filtrates selectively altered amino acid and energy metabolism in ways that align with established mechanisms of *C. difficile* pathogenesis. Proline, a primary substrate for Stickland fermentation and a determinant of *C. difficile* growth and toxin regulation, was reduced following filtrate exposure, consistent with impairment of a key energy and virulence pathway [32,33]. Fumarate and succinate, metabolic intermediates that accumulate during dysbiosis and can be exploited by *C. difficile* to support expansion and biofilm formation, were also depleted, indicating a shift away from a nutrient milieu that favours pathogen proliferation [34,35]. Conversely, tryptophan levels increased, a change consistent with activation of microbiota-dependent immunoregulatory pathways (e.g., AhR signalling) that promote mucosal homeostasis and attenuate inflammation [36]. Together, these shifts suggest that faecal filtrates impair *C. difficile* by simultaneously disrupting pathogen energy acquisition and reducing virulence-associated metabolism while promoting host-directed immunoregulatory programmes.

Spatially resolved 3D OrbiSIMS imaging revealed heterogeneous metabolite distributions within *C. difficile*, providing the first direct visualisation of metabolic compartmentalisation in this pathogen [13]. Proline and succinate were widely distributed, whereas fumarate showed localised accumulation, consistent with microenvironmental specialisation observed in anaerobic fermentation [37]. PI and PE lipids, along with phosphate ions, were detected on the cell surface, underscoring their roles in membrane architecture.

These spatial and functional insights extend beyond rCDI, establishing a methodological framework for probing microbial metabolic organisation. Importantly, sterile faecal filtrates alone modulated bacterial lipid pathways and delivered metabolites with host-directed effects. Exogenous PI application significantly attenuated epithelial barrier disruption caused by *C. difficile* toxins, DSS, and *Staphylococcus aureus* enterotoxin B, consistent with prior reports that PI lipids stabilise epithelial membranes and modulate inflammatory signalling [19,29,38].

Collectively, our findings provide direct functional evidence that filtrate-derived metabolites are active effectors that impair pathogen fitness while safeguarding host tissues. They also align with previous work linking altered lipid metabolism to antimicrobial resistance, highlighting the bacterial membrane as a therapeutic target [26,31,39].

Limitations of this study include small sample size, reliance on in vitro co-culture systems, which cannot fully recapitulate host-microbiome interactions such as immune responses and epithelial signalling. Validation in animal models and humanised systems, including organoids and organ-on-chip models, will therefore be essential [40,41,42,43]. Furthermore, all patients in this study achieved clinical resolution after FMT, precluding comparison of metabolic differences between responders and non-responders. Larger studies, including FMT failures, will be critical to identify predictive biomarkers of therapeutic success.

## 4. Materials and Methods

### 4.1. Bacterial Strains and Culture Conditions

Two toxigenic Clostridioides difficile strains were used: a low-toxin producer (CD630) and a high-toxin producer (VPI 10463), obtained from ATCC (Manassas, VA, USA). Strains were cultured in Brain Heart Infusion (BHI) broth (Oxoid, Basingstoke, UK) supplemented with 0.5% yeast extract (Oxoid, Basingstoke, UK) and 0.1% cysteine (ThermoFisher Scientific, Loughborough, UK), 250 µg/mL D-cycloserine and 8 µg/mL cefoxitin (Oxoid, Basingstoke, UK). Cultures were incubated anaerobically at 37 °C overnight in a workstation (Don Whitley Scientific, Bingley, UK) under an atmosphere of 10% CO_2_, 10% H_2_ and 80% N_2_. All media were pre-reduced before use.

### 4.2. Preparation of Donor-Derived Faecal Filtrates for 3D OrbiSIMS

Frozen faecal samples were obtained from healthy donors (n = 4; 2 male, 2 female; mean age 33.8 ± 2.4 years) enrolled in the Edmonton FMT programme and screened according to published guidelines [44]. Samples were stored at −80 °C until processing. Faecal filtrates were prepared by homogenising 100 mg of thawed faeces in sterile PBS at a ratio of 1:4 (*w*/*v*), centrifuging at 10,000× *g* for 10 min at 4 °C, and filter-sterilising the supernatant through a 0.22 μm membrane. Sterility of the faecal filtrates was confirmed by plating aliquots on Tryptic Soy Agar (TSA) for aerobic bacteria and (BHI) agar for anaerobic bacteria, followed by incubation at 37 °C for 48 h; no microbial growth was observed in either condition. Sterile faecal filtrate was added to C. difficile bacterial cultures 10% *v*/*v* and incubated for 4 h at 37 °C. Each faecal filtrate sample was spotted and analyzed in technical triplicate to account for variability in the instrument and sample preparation. A 1 µL aliquot of each sample was transferred to 8-well plates, freeze-dried overnight, and stored in liquid nitrogen until 3D OrbiSIMS analysis. As a control, sterile PBS processed in the same way as faecal filtrates (without biological material) was analysed to monitor potential background signals.

### 4.3. Metabolite Preparation and Treatment

The following metabolites were used in this study: phosphatidylinositol (PI) (18:0/20:4) (CAS Number: 1331751-28-0), lysophosphatidylinositol (LPI) (20:4/0:0) (CAS Number: 1246430-04-5), and PI (16:0/18:1(9Z)) (CAS Number: 50730-13-7), all obtained from Avanti Polar Lipids (Alabaster, AL, USA) or Sigma-Aldrich (St. Louis, MO, USA) in 100 μg quantities, ≥99% purity. Each metabolite was initially dissolved in sterile methanol at 10 mM, sonicated for 30 s, and then diluted into sterile BSA/EGTA solution (1% bovine serum albumin [BSA] in phosphate-buffered saline [PBS] containing 1 mM ethylene glycol-bis(β-aminoethyl ether)-N,N,N′,N′-tetraacetic acid [EGTA]) to prepare working solutions. The final lipid concentrations used for cell treatment were 10 µM, with a solvent ratio of <0.5% methanol in the final mixture. Prior to treatment, the cell culture media were removed and replaced with the metabolite/BSA/EGTA mixture, which was incubated with the cells for 15 min at 37 °C. The mixture was then aspirated, fresh media were replenished, and barrier integrity measurements were conducted 24 h post-treatment.

### 4.4. Preparation of Caco-2 Transwell Monolayers

To assess intestinal epithelial barrier permeability, triplicates of ~5000 Caco-2/TC-7 human colon adenocarcinoma (Sigma-Aldrich) cells/well were seeded onto 96-well Corning™ HTS Transwell™ 96-Well Permeable Support Systems (cat. no. 10175562, Fisher Scientific) and cultured for 4 weeks, allowing for cell differentiation, polarisation and formation of continuous monolayer. Dulbecco’s Modified Eagle Medium (DMEM; cat. no. 11995-065, Gibco, Thermo Fisher Scientific, Waltham, MA, USA) was supplemented with 20% fetal bovine serum (FBS, cat. no. 10500-064, Thermo Fisher Scientific), Penicillin-Streptomycin (cat. no. 15070-063, ThermoFisher) and non-essential amino acids (cat. no. 11140-050, ThermoFisher). Cells were verified as being free from mycoplasma contamination. The incubator was set at 37 °C with 95% relative humidity. Cells were split by washing with PBS (cat. no. 14040-091, ThermoFisher), followed by adding 1 mL trypsin-EDTA (0.25%) (cat. no. 25200-056, ThermoFisher) and incubating for 5 min. DMEM (three times the volume of trypsin) was then added to neutralise the enzyme. The suspension was transferred into 15 mL falcon tubes, centrifuged (4 min at 1200 RPM), and the resulting pellet was resuspended in 1 mL fresh medium for sub-culturing. Cells were mixed gently by pipetting and seeded into sterile dishes at the required density. Plasmocin prophylactic (25 mg, cat. no. ant-mpp, InvivoGen, San Diego, CA, USA) was added to Eagle’s Minimum Essential Medium (EMEM; Gibco, Thermo Fisher Scientific) used for SK-MEL-2 cell line maintenance, where necessary, to prevent mycoplasma contamination.

### 4.5. Barrier Integrity Measurements and Toxin Exposure

To assess intestinal barrier function, Caco-2 cells (~5000 cells/well) were seeded onto the Transwell and maintained under standard incubation conditions (37 °C, 95% humidity, 5% CO_2_). Media in both the upper and lower chambers were replaced every three days. Full confluency and functional barrier formation were typically achieved within 2–3 weeks, at which point FITC-dextran (Sigma-Aldrich, St. Louis, MO, USA; 10 µM/mL) fluorescence was measured (48, 72, and 96 h) following the manufacturer’s guidelines, using a microplate reader (Multiskaan-SkyHigh, Thermofisher, Waltham, MA, USA) and MARS data analysis software v. 3.01R2 (BMG-LABTECH, Ortenberg, Germany).

For the corresponding metabolites, 100 µL of each lipid metabolite (10 µM) was added to triplicate wells and incubated for 15 min at 37 °C at 5% CO_2_. Each well was aspirated and 90 µL of no-phenol red DMEM (with 20% FBS) (Thermofisher) was added to each well. Microbial toxin (10 µL, see below) was added to each well to create a final volume of 100 µL per well.

Microbial toxins TcdA and TcdB from C. difficile (50 pg/mL; Native Antigen Company, Kidlington, UK), Pseudomonas exotoxin A (Cambridge Bioscience; Cambridge, UK; 3.5 µg/mL), 4% dextran sulfate sodium (DSS; Sigma), and Staphylococcus aureus toxin (Cambridge Bioscience; 2.5 µg/mL) were added to the corresponding wells in triplicate and incubated for 24 h.

### 4.6. 3D OrbiSIMS Data Analysis

Secondary ions of interest were automatically selected using a threshold of 0.1% of the base peak intensity. The minimum peak of 1000 was applied to distinguish true peaks from noise, determined via peak search in Surfacelab software v 7.1 (ION-TOF, GmbH, Münster, Germany). Peak intensities were normalised to total ion counts to account for variability in sample spotting and instrument response; all reported values represent relative metabolite abundance rather than absolute concentrations. Depth profile spectra were exported as .TXT files from IONTOF SurfaceLab 7 and aligned within a 2-ppm *m*/*z* window using Molecular Formula Prediction software, SIMS-MFP v 1.1 (Univeristy of Nottingham, Nottingham, UK) implemented as an in-house MATLAB script (R2020a, The MathWorks, Inc., Natick, MA, USA) [45]. Chemical filtering (SIMS MFP) enabled assignment of peaks in both positive and negative ion spectra, with putative metabolite identities based on accurate mass (<2 ppm deviation). Although MS/MS spectra or authentic standards were not employed, the high mass accuracy provides strong support for these assignments. Background peaks were removed prior to statistical analysis, and data were Pareto scaled [46].

Principal Component Analysis (PCA) was performed in SIMCA^®^ 13.0.3 (Umetrics, Umeå, Sweden). Orthogonal Partial Least Squares Discriminant Analysis (OPLS-DA) was applied as a supervised multivariate model to assess analytical performance and identify metabolic clustering or separation [47].

### 4.7. rCDI Participant Stool Samples

Stool samples from 12 patients with rCDI successfully treated with a single treatment in a randomised controlled trial comparing capsule- and colonoscopy-delivered FMT [14]. The cohort included both male and female participants (mean age 63 ± 18.9 years); six received FMT via colonoscopy and six via oral capsules. Stool samples were collected prior to and 12 weeks after FMT and were immediately frozen at −80 °C until analysis and chosen based on availability.

### 4.8. Statistical Analysis

All statistical analyses were performed using GraphPad Prism v10.1.2 software (GraphPad Software, San Diego, CA, USA). Differences between the two groups were assessed using unpaired *t*-tests. For experiments involving more than two factors, two-way analysis of variance (ANOVA) followed by Tukey’s multiple comparisons test was applied. Data are presented as mean ± standard deviation (SD) from biological triplicates. A *p*-value ≤ 0.05 was considered statistically significant. Exact n values, SDs, and test parameters are reported in the corresponding figures and legends. For OrbiSIMS data analysis, statistical significance was assessed by Student’s *t*-tests and variable importance in projection (VIP) scores from OPLS-DA. Metabolites were considered significantly altered if they met all criteria: *p*-value threshold of <0.05, VIP > 1, and a fold change (FC) of ≥2, with false discovery rate (FDR) control applied [48]. Metabolite annotation and pathway enrichment were conducted using MetaboAnalayst 6.0 and the Kyoto Encyclopaedia of Genes and Genomes (KEGG) database [49,50,51].

## 5. Conclusions

This study offers the first mechanistic insight into how faecal filtrates restore metabolic balance in rCDI. Through spatial metabolomics, we revealed shifts in amino acid and energy metabolism and enrichment of phosphatidylinositol lipids that both impair *C. difficile* and preserve gut barrier integrity. Spatial profiling illustrated metabolic compartmentalisation within *C. difficile*, advancing methodological frontiers. Crucially, sterile filtrates alone disrupted pathogen energy and lipid metabolism and delivered cytoprotective PI lipids, highlighting a dual mechanism of action: reducing pathogen fitness while safeguarding host barrier function. While in vitro limitations and absence of non-responder comparisons warrant cautious interpretation, our findings support development of metabolite cocktails or defined microbial consortia, validated in humanised gut models, as promising metabolically informed therapies for rCDI and microbiome-driven disorders.

## Figures and Tables

**Table 1:** Metabolites significantly enriched in pre-FMT stool samples of rCDI patients. (n = 12), showing fold change > 2, *p* < 0.05 and VIP > 1.

Metabolite	Fold Change (FC)	log2(FC)	*p*-Value	VIP
**(S)-malate**	37.276	5.2202	0.002504	1.051211
**cis-aconitate**	10.632	3.4103	0.000248	1.083543
**citrate**	72.491	6.1797	0.000338	1.0771
**Deoxy uridine**	9.5446	3.2547	0.000632	1.070974
**D-glucopyranose**	327.66	8.3561	0.000315	1.079193
**Guanine**	19.083	4.2543	0.000704	1.070274
**Lysine**	734.13	9.5199	0.000248	1.083333
**Phenylalanine**	10.308	3.3657	0.000886	1.067918
**Proline**	49.838	5.6392	0.000236	1.085902
**Thymidine**	5.8103	2.5386	0.000113	1.058325
**Thymine**	14.014	3.8088	0.000315	1.079273
**Tryptophan**	55.978	5.8068	0.000315	1.08043
**Uracil**	13.239	3.7267	0.000406	1.076506
**Uridine**	72.225	6.1744	0.000248	1.081858

**Table 2:** Metabolic pathways significantly altered 12 weeks after FMT.

Pathway	FDR-Adjusted *p*-Value
Glyoxylate and dicarboxylate metabolism	0.000359
Glycerophosphoinositol pathway	0.000645
Valine, leucine and isoleucine biosynthesis	0.004422
Phenylalanine, tyrosine and tryptophan biosynthesis	0.004422
Alanine, aspartate and glutamate metabolism	0.013322

## Data Availability

The data presented in this study are openly available in [Figshare] at https://doi.org/10.6084/m9.figshare.28878800, reference number [28878800]; accessed on 24 April 2025.

## Supplementary Materials

The following supporting information can be downloaded at: https://www.mdpi.com/article/10.3390/ijms262211221/s1.
