# Supplementary material for: Untargeted Metabolomics Identifies Faecal Filtrate-Derived Metabolites That Disrupt Clostridioides difficile Metabolism and Confer Gut Barrier Cytoprotection
Source: Int J Mol Sci. 2025 Nov 20;26(22):11221. doi: 10.3390/ijms262211221 (PMC12653063; doi:10.3390/ijms262211221)
Supplement: Supplementary file 1 [file ijms-26-11221-s001.zip › ijms-3968816-supplementary.pdf]

Supplementary Data

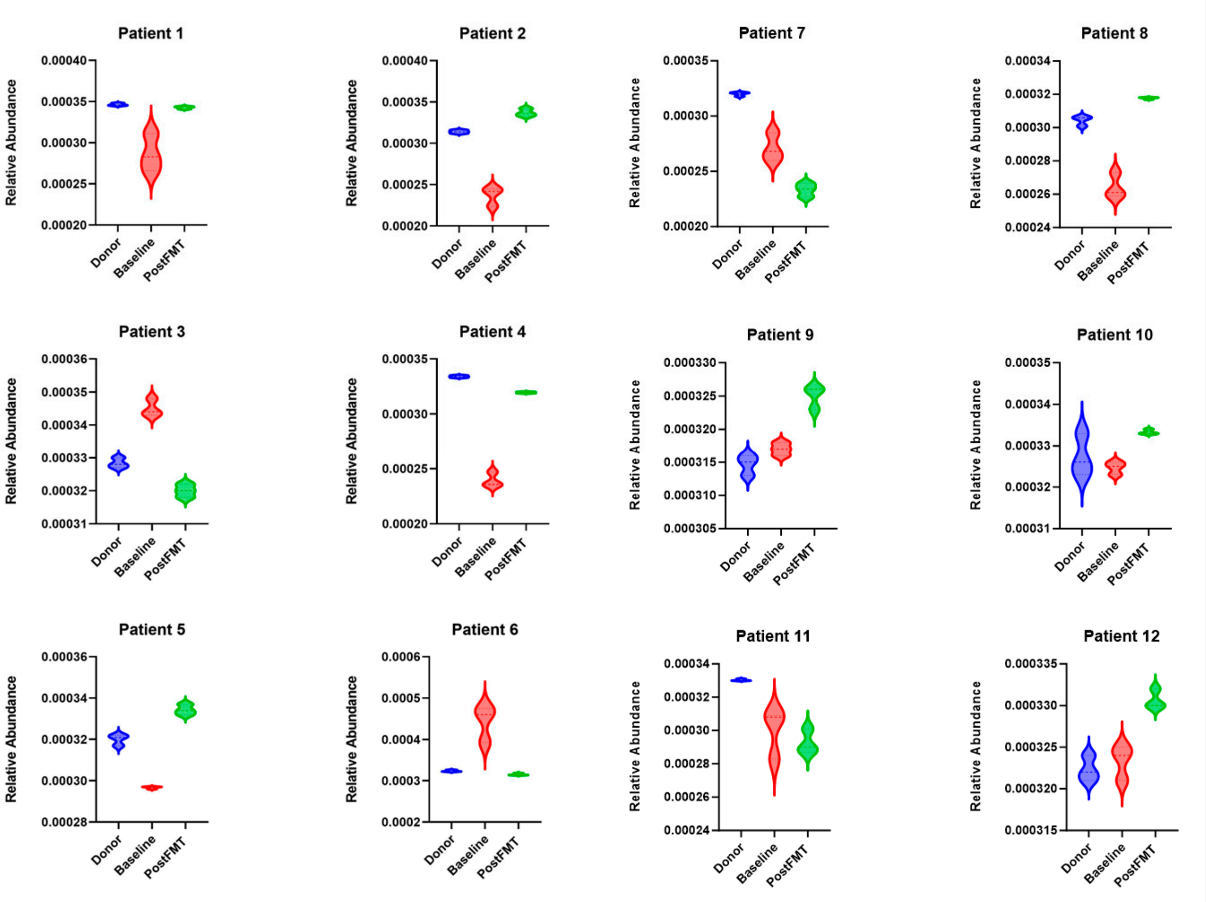

Supplementary Figure S1. Violin plots of faecal metabolite abundance in rCDI patients pre- and post-FMT. Relative abundance of identified metabolites for 12 patients at baseline (pre-FMT, red), 12 weeks post-FMT (green), and donor samples (blue). Each plot represents an individual patient. Post-FMT profiles exhibited a general trend toward normalisation, with metabolite levels shifting closer to those of healthy donors. Data were analysed using GraphPad Prism v10. FMT denotes faecal microbiota transplantation, rCDI recurrent *Clostridioides difficile* infection.

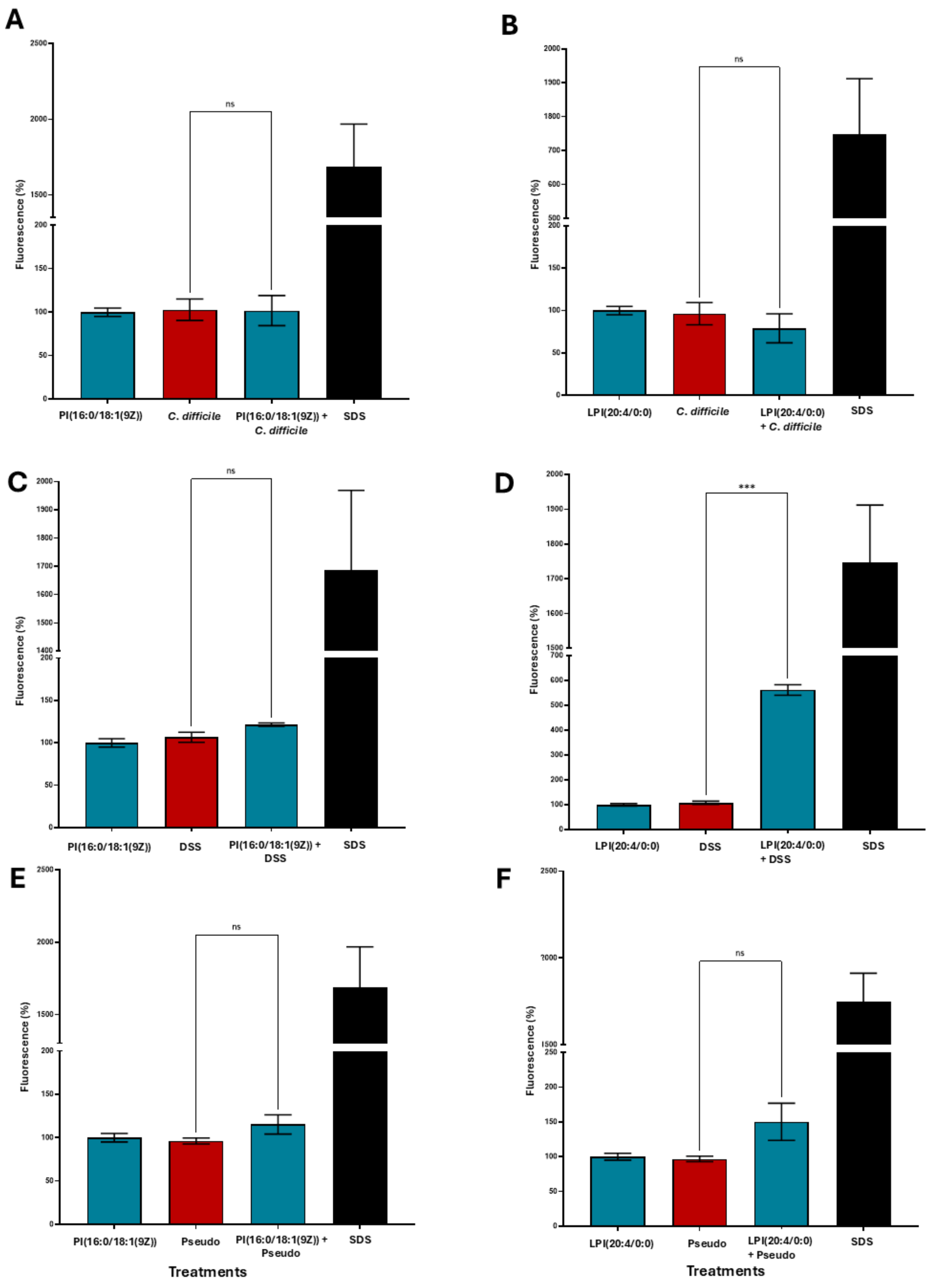

Supplementary Figure S2. Around 5000 Caco-2 cells were seeded in biological triplicates (n=3) into transwells. After 2-weeks, IEC confluency and barrier integrity was measured using transepithelial electrical resistance (TEER) measurements. Once the barrier was established, the wells were transfected with their corresponding siRNA (siCFTR or siControl). After 24h, the metabolite was added. 15mins later, the microbial toxin insult (*Clostridioides difficile,* toxins A&B), *DSS* and *Pseudomonas aeruginosa* (enterotoxin A) were added to the corresponding wells. Fluorescence measurements were taken at 48h. Results were expressed as mean ± SEM. Statistical significance is indicated by asterisks (***p < 0.001), while "ns" denotes no significant difference. Panels A and B show that neither PI (16:0/18:1(9Z)) nor LPI (20:4/0:0) significantly altered fluorescence when combined with *C. difficile* toxins. Similarly, Panels C, E, and F demonstrate that PI (16:0/18:1(9Z))

and LPI (20:4/0:0) had no significant effect when paired with DSS or *Pseudomonas aeruginosa* enterotoxin A, respectively. In contrast, Panel D reveals a significant increase in fluorescence when LPI (20:4/0:0) was combined with DSS, suggesting a synergistic disruption of epithelial barrier integrity under this condition.
